# The Emerging Role of the Cytoskeleton in Chromosome Dynamics

**DOI:** 10.3389/fgene.2017.00060

**Published:** 2017-05-19

**Authors:** Maya Spichal, Emmanuelle Fabre

**Affiliations:** ^1^Department of Genetics, University of North Carolina, Chapel HillNC, United States; ^2^Equipe Biologie et Dynamique des Chromosomes, Institut Universitaire d’Hématologie, CNRS UMR 7212, INSERM U944, Hôpital St. Louis 1Paris, France

**Keywords:** nucleus, cytoskeleton, chromosomes, dynamics, yeast

## Abstract

Chromosomes underlie a dynamic organization that fulfills functional roles in processes like transcription, DNA repair, nuclear envelope stability, and cell division. Chromosome dynamics depend on chromosome structure and cannot freely diffuse. Furthermore, chromosomes interact closely with their surrounding nuclear environment, which further constrains chromosome dynamics. Recently, several studies enlighten that cytoskeletal proteins regulate dynamic chromosome organization. Cytoskeletal polymers that include actin filaments, microtubules and intermediate filaments can connect to the nuclear envelope via Linker of the Nucleoskeleton and Cytoskeleton (LINC) complexes and transfer forces onto chromosomes inside the nucleus. Monomers of these cytoplasmic polymers and related proteins can also enter the nucleus and play different roles in the interior of the nucleus than they do in the cytoplasm. Nuclear cytoskeletal proteins can act as chromatin remodelers alone or in complexes with other nuclear proteins. They can also act as transcription factors. Many of these mechanisms have been conserved during evolution, indicating that the cytoskeletal regulation of chromosome dynamics is an essential process. In this review, we discuss the different influences of cytoskeletal proteins on chromosome dynamics by focusing on the well-studied model organism budding yeast.

## Overview

As its name says, the cytoskeleton is the skeleton of the cell that is partially responsible for its form and structure. The cytoskeleton’s principal proteins can form polymers. The cytoskeleton consists of tubulin (that forms microtubules), actin (that forms microfilaments, also called actin cytoskeleton) and lamin (the basic subunit of intermediate filaments). Due to the rapid assembly and disassembly of cytoskeletal proteins the cytoskeleton is very dynamic and necessary for multiple cellular processes like cell division and motility. In addition to its role in cell motility, many studies support links between the cytoskeleton and chromosome motion. For instance, during meiosis, cytoskeleton dependent chromosome movements are important during prophase and promote correct genetic inheritance. The role of the cytoskeleton in chromosome movement during the mitotic cell cycle has, however, only recently been investigated. In this review, we discuss the role of the cytoskeleton in chromosome movement during the mitotic cell cycle by concentrating on the well-studied unicellular eukaryote model organism yeast *Saccharomyces cerevisiae*. Chromosome dynamics rely on several parameters, including chromatin architecture and non-random chromosome organization inside the nuclear space. Chromosome motion depends to a large extent on the accessibility of the chromatin fiber by different regulators, which also influence DNA functional transactions including transcription, replication and DNA repair. For a better understanding of chromosome motion, we will first characterize the different levels of chromatin architecture. We will describe what is known in terms of yeast chromosome organization and discuss its impact on chromosome motion. A particular focus will be given to cytoskeletal proteins found inside the nucleus and their role in chromosome dynamics.

## Chromatin Structure Influences Chromatin Motion

The DNA molecule is a flexible fiber that is condensed by octamers of proteins, the nucleosomes, that form a first level of compaction with a regular spacing of 1–165 bp along the DNA. This forms a fiber of about 10nm that can be observed as ‘beads on a string’ in electron microscopy (Lavelle et al., 2010). The interaction of the DNA molecule with its structuring proteins constitutes chromatin. Each nucleosome octamer is usually composed of two tetramers of H2A, H2B, H3, and H4 histones wrapped by about 147 pb ± 2 bp of DNA. The N-terminal regions of the histone proteins are not strictly bound to DNA and form amino acid tails that extend into the nucleoplasm. These N-terminal ends are extensively post-translationally modified by acetylations, methylations, and others like phosphorylations, ubiquitinations, and sumoylations (Jaiswal et al., 2016). These post-translational modifications regulate the interaction of the nucleosomes with DNA, determine the level of accessibility of the DNA for other regulatory proteins and thus serve as a first control for the regulation of DNA compaction and stiffness. There is also a wide number of histone variants that serve in different signaling functions and change the compaction of the fiber through a yet not discovered mechanism (Weber and Henikoff, 2014). The association of various histones and the type of modification of the histone tails is very dynamic and depends on numerous factors like the genomic position with which they are associated, the cell cycle stage or the type of DNA damage the chromatin can face (Swygert and Peterson, 2014). Initially proposed in the lab of David Allis (Jenuwein and Allis, 2001), the ensemble of the regulation of DNA by histones, called ‘histone code’, is the first layer of genome organization. It is also a crucial parameter in local genome dynamics.

Interestingly, histones appear to be determinants for chromosome dynamics not only by their posttranslational modification but also by their abundance. It has been known for some time that global chromosome dynamics increase if multiple DNA double strand breaks are generated (Miné-hattab and Rothstein, 2012; Dion et al., 2012; Seeber et al., 2013). Hauer et al. (2017) describe that cellular histone levels can drop by 20–40% in the presence of DNA damage. This histone loss is proteasome-mediated and necessitates both the DNA damage checkpoint and the INO80 chromatin remodeler (Hauer et al., 2017). It is proposed that histone loss results in chromatin decompaction, which in turn could provoke an increased flexibility of the chromatin fiber leading to higher chromosome dynamics. Interestingly, previous work by Verdaasdonk et al. (2013) also studied the spatio-temporal organization of the chromatin fiber in the case of histone H3 depletion. In this study, the Rouse polymer model is used to analyze the physical properties underlying chromosome fluctuations. In the Rouse model, it is assumed that the monomers are connected together by springs of stiffness Ks, with an average distance between consecutive monomers equal to b. In the basic Rouse model, b corresponds to the persistence length Lp (or Kuhn length, which equals two times Lp). In this case, Ks = kB T/Lp^2^ (with kB the Boltzmann constant and T the temperature in Kelvin). In the case of histone depletion, the model predicts a decrease of distance b (or Lp), and an increase of the stiffness Ks between two beads, as opposed to a decompaction. It should be observed that if the distance b (or Lp) decreases, then the stiffness Ks increases, but it is not the stiffness of the chain in the sense of a resistance to bending which requires an energy potential between three consecutive monomers, whereas the energy of the Rouse spring depends only on the distance between two consecutive monomers.

Thus, the results obtained in these two studies (Verdaasdonk et al., 2013; Hauer et al., 2017) therefore point the physical changes of the chromatin fiber upon histone depletion, but differ in their interpretation. Additional methods, such as super resolution microscopy, are expected to shed light on chromatin physical properties changes upon histone depletion.

Of note, as it will be detailed below, chromosome tethering to the nuclear envelope or to the SPB is an important regulator of chromosome motion, it is therefore critical to consider this parameter when studying chromatin fiber properties (Heun et al., 2001; Verdaasdonk et al., 2013; Strecker et al., 2016; Spichal et al., 2016). It is interesting that chromatin decompaction can also change chromatin localization in mammalian cells in a transcription independent manner (Therizols et al., 2014).

Because chromatin structure has a role in dynamic chromatin function, a large part of the past literature has studied a second level of chromatin organization, which is the 30 nm fiber. Initially, 30 nm fibers were discovered *in vitro*, by increasing the ionic strength of the solvent from chromatin observation by electron microscopy (Thoma et al., 1979). 30 nm fibers were described as the stacking of the 10 nm fiber into one of two models, solenoid or zigzag of about 30 nm. *In vivo* biological reality of the 30 nm fiber is uncertain. A fiber of 32 nm in diameter has only been found in avian erythrocytes with a helix of two starting points (Scheffer et al., 2011). However, 10nm and not 30 nm fibers were found in various other cell types (Ahmed et al., 2010; Maeshima et al., 2010; Fussner et al., 2011; Nishino et al., 2012; Hsieh et al., 2015). Chromatin *in vivo* is therefore possibly found in the 10 nm state, or in a state in between the 10 nm and the 30 nm fiber, for most of the cell cycle, facilitating dynamic DNA communications.

Understanding chromosome architecture has further gained from a large number of studies based on chromosome conformation capture (3C) experiments. This technique, first published in 2002 by Job Dekker and Nancy Kleckner, showed the 3D conformation of the budding yeast chromosome III through the characterization of the inter and intrachromosomal interactions (Dekker et al., 2002). A frequent cutter digests chromosomes, which are then crosslinked by formaldehyde through their protein interactions and the resulting fragments are ligated in dilute conditions. Due to the crosslinking, fragments from different chromosomal regions can ligate and are analyzed by PCR using primers positioned at certain linear genomic distances. Since 2002, several derivatives of this technique have been developed and used on different organisms and cell types (Lieberman-Aiden et al., 2009; Duan et al., 2010; Dixon et al., 2012; Hou et al., 2012; Sexton et al., 2012; Nora et al., 2012; Kalhor et al., 2012; Cagliero et al., 2013; Hsieh et al., 2015). A number of physical parameters that characterize the chromatin fiber can be extracted from these studies including the persistence length (lp) of chromatin, which describes its stiffening or flexibility in nm and the compaction (c) in bp/nm (contour length of the fiber, c would be infinitely large in the case of an unconstrained fiber). In budding yeast, the chromatin persistence length was found to range between 0, an unexpectedly small value, and 200 nm using different experimental techniques and c values are found of around ∼50 bp/nm (Bystricky et al., 2004; Dekker, 2008; Hajjoul et al., 2013). Despite discrepancies, this makes yeast chromatin appear flexible and open, with likely consequences on chromatin motion.

## Chromatin Motion is Characterized By Subdiffusion

Movement can be characterized by its energy and directionality. Molecules can adopt four different types of motion: random or Brownian movement, confined diffusion, anomalous diffusion and directed movement. Different studies have shown that different metabolic processes like DNA replication and DNA repair require specific chromosome movements (Heun et al., 2001; Dion et al., 2012; Miné-hattab and Rothstein, 2012; Seeber et al., 2013; Spichal et al., 2016). Traditionally, there are two different methods to measure chromosome movement. The first and more indirect method is the measurement of fluorescently labeled and chromatin-bound proteins after fluorescence recovery after photobleaching (FRAP). This method can measure how fast the fluorescence of a chromatin-bound protein is recovered. Recovery happens either by diffusion of chromatin bound by the labeled protein or by free diffusion of the labeled protein in the nucleoplasm, in which case this method is flawed. Fluorescently labeled histones are commonly used (Gerlich and Ellenberg, 2003; Walter et al., 2003). By using FRAP, chromatin diffusion can often only be described as fast or slow according to a reference and different types of diffusion cannot be determined (see diffusion types below).

The second method is a more direct technique to measure chromosome movement and uses the fluorescent repressor operator systems (FROS) that allows fluorescently labeled proteins to bind to a specific DNA sequence in order to follow the labeled chromosome locus by microscopy. In FROS, a bacterial tetracycline (tet) or lactose (lac) operator sequence is integrated into the genome. The corresponding repressor protein is fused to fluorescent proteins. The tight interaction of the repressor and the operator allows the chromosomal locus to be detectable and can be tracked by fluorescence microscopy (Lassadi and Bystricky, 2011). To avoid exogenous DNA insertions into the genome, less intrusive alternatives are being developed, including TALE (transcription activator-like effector) and inactive CRISPR (Clustered regularly interspaced short palindromic repeats) proteins (Chen et al., 2013; Zhang et al., 2014). Fused to fluorescent proteins, these proteins offer the advantage to label specific DNA sequences, but because low signal to noise ratio (SNR), only repeated sequences (telomeres or heterochromatin) have been detected so far. These methodologies therefore await improvement in SNR to detect non-repeated loci. When detected, the labeled chromosomal locus can be followed by fluorescence imaging in living cells over time. The mean square displacement (MSD) of a given labeled locus can be calculated, which is a measure between the deviation of a position of the chromosome locus and a reference position. The MSD can be regarded as the amount of space the observed particle, here a labeled chromosomal locus, has travelled in its system, the nucleus.

$$\text{MSD}(\partial )=[x(t)-x{\left(t+\partial \right)}^{2}]+[y(t)-y{\left(t\partial \right)}^{2}]+\left[z(t)-z{\left(t+\partial \right)}^{2}\right]$$

Where x, y, and z are the spatial coordinates of the locus in three dimensions to measure and ∂ is the difference in time (t). The MSD measures the mean square displacement for a particle for a given time interval in a trajectory. In the case of an isolated particle, thermal agitation animates the molecules to a continuous and random movement called Brownian motion. This movement is diffusive and characterized by a linear variation of the MSD in time. The below described formula characterizes the MSD in three dimensions:

MSD(t)=6⁢ D⁢ t

where D is the diffusion coefficient. Many of the current analyses, however, are made in two dimension excluding the z-axis due to the loss of resolution from the nature of the point spread function, and MSD (t) = 4 D t, then applies. Diffusion is not normal when it takes place in a complex fluid medium that might contain random or fractal obstacles as it is the case in the interior of the cell that is crowded with macromolecules, or when it is not an isolated particle, as it is the case for a locus integrated in the chromosome.

In these cases, the MSD has a non-linear relationship to time and its behavior is anomalous.

$$\text{MSD}\left(t\right)=\Omega \,\;t^{\alpha }$$

**FIGURE 1:**
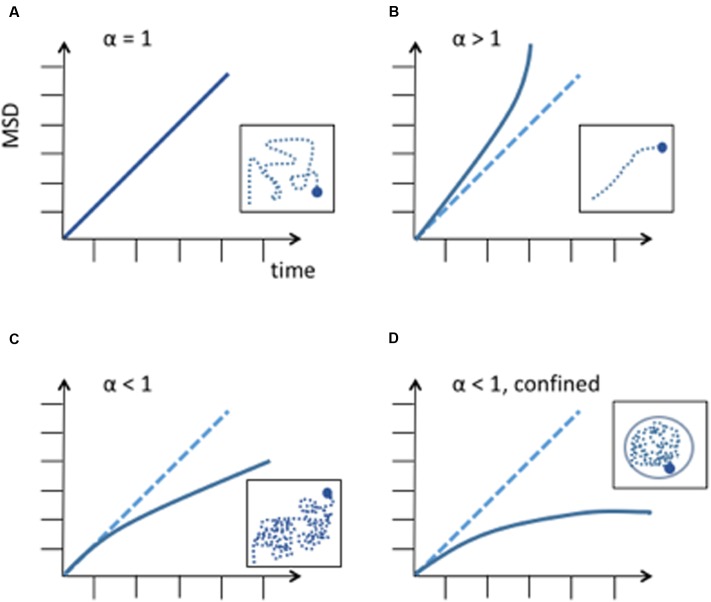
**Different diffusion behaviors of a particle.** MSD is expressed in μm^2^. **(A)** Random diffusion of a particle, this corresponds to Brownian motion. **(B)** Anomalous diffusion described by a power law with α > 1. This corresponds to super-diffusion or directed motion. **(C)** Anomalous diffusion described by a power law with α < 1, subdiffusion. A subdiffusive behavior has been observed in most chromosome dynamics studies. **(D)** Subdiffusion in a confined space. When a chromosome locus encounters a boundary, the curve of the MSD reaches a plateau. Differences in measurements can be explained by chromosome loci position along the chromosome and to its tethering to nuclear structures, like the nuclear envelope or nuclear microtubules.

Where, Ω is a coefficient and α is the exponent of anomalous diffusion. In the case of a subdiffusive behavior α < 1, in the case of linear diffusion α = 1, and in the case of superdiffusion α > 1. The nature of diffusion significantly changes the way a particle explores its available space and the time to reach a specific destination (**Figure 1**). Therefore, it is important to accurately agree on the nature of diffusion. Once the type of diffusion has been determined the MSD can be calculated for a particle’s trajectory and further information can be revealed by best fitting the MSD curves from collected trajectories. The height of the curve gives information about the amount of space the chromosomal locus has explored and the time to reach a specific position while the shape of the curve reveals the nature of the movement (Guérin et al., 2012) (**Figure 1**). If the MSD curve reaches a plateau, the particle’s trajectory is confined and the curve’s height is correlated to the confinement radius *R*_c_.

$$R_{c}=\frac{5}{4}\sqrt{{\text{MSD}}_{\text{plateau}}}$$

Chromosome movement is energy-dependent and requires ATP (Heun et al., 2001). Studies of chromosome movement in eukaryotes and prokaryotes have suggested that chromosomes either adapt to confined diffusion and/or anomalous diffusion. Confined diffusion is understood as a molecule freely diffusing but confined by the nuclear periphery, other obstacles or spatial exclusion due to other macromolecules (Marshall et al., 1997; Heun et al., 2001; Gartenberg et al., 2004; Bystricky et al., 2005; Levi et al., 2005; Bronstein et al., 2009; Miné-hattab and Rothstein, 2012). On the other hand, chromosomal loci were found to be constrained in their trajectory and followed an anomalous subdiffusion behavior in a number of other studies (Levi et al., 2005; Thakar and Csink, 2005; Cabal et al., 2006; Espeli et al., 2008; Bronstein et al., 2009; Weber et al., 2012; Hajjoul et al., 2013; Javer et al., 2013; Saad et al., 2014). For a simple Rouse chain, it is the expectation that the exponent in an MSD plot will be on the order of 0.6 in a good solvent. Thus, from the polymer perspective, it is predicted that a locus in a chromosome (a spot on the chain) will not behave like a diffusing particle. Interestingly, anomalous subdiffusion has also been found in prokaryotes although they do not possess a nucleus and their chromosomes are not organized by nucleosomes but by proteins that are similar to histones (Espeli et al., 2008; Macvanin and Adhya, 2012; Weber et al., 2012). The finding that the exponents are very close to the expected Rouse model in both prokaryotes and eukaryotes is thus predicted due to the physical properties of the chain, and not other factors relating to nuclear confinement and makes subdiffusive behavior a universal characteristic of a chromosomal locus motion. Of note, while yeast telomeres were generally found to have diffusion coefficients ranging from 1.5 to 7 10^-3^ μm^2^s^-1^ (Heun et al., 2001; Cabal et al., 2006; Hajjoul et al., 2013; Spichal et al., 2016), mammalian telomere diffusion coefficients were recently found in the range of 0.28-1.1 10^-3^ μm^2^s^-1^ (Bronshtein et al., 2015; Lottersberger et al., 2015). Thus, speed and properties of telomeres dynamics of large and small genomes, is unexpectedly comparable. Furthermore, as we will discuss below, directed dynamic chromosome movement are also described in some studies, involving the Linker of the Nucleoskeleton and Cytoskeleton (LINC) complex and cytoskeleton counterparts.

## Motion of A Given Chromosomal Locus Depends on its Spatial Environment

**FIGURE 2:**
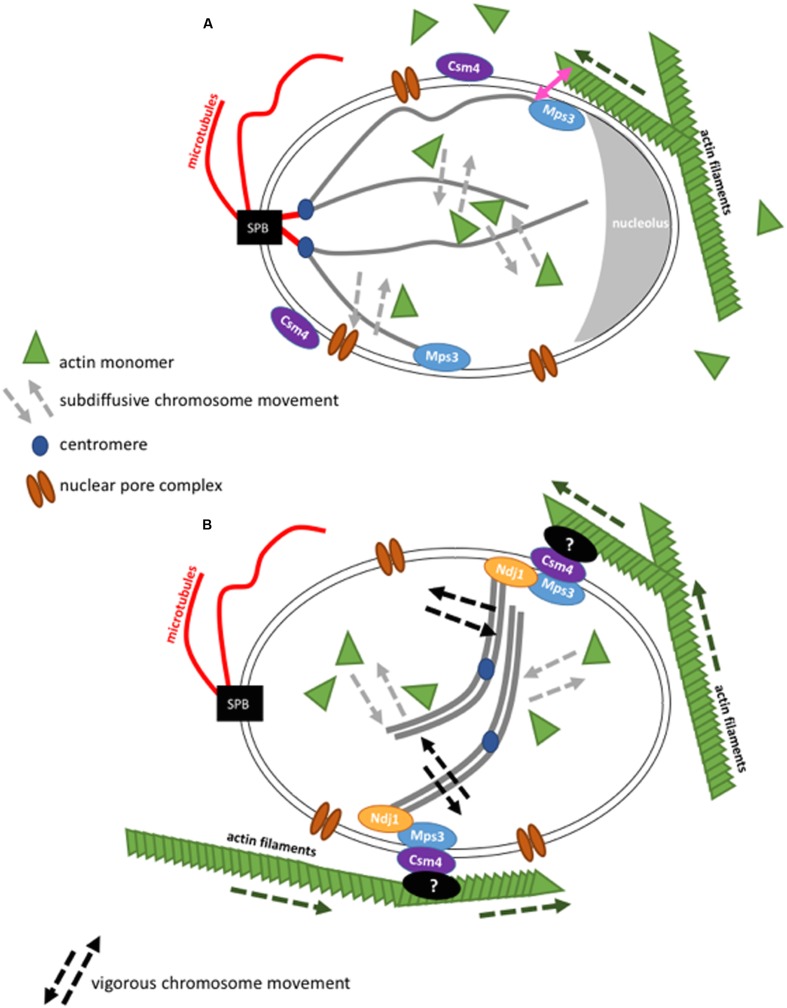
**Schematic representation of the interaction of actin with chromosome movement in the cell nucleus in budding yeast.** Figures are not drawn to scale. **(A)** During the mitotic cell cycle, budding yeast chromosomes are arranged in a Rabl configuration. In interphase, the SPB is found opposite to the nucleolus. In the cytoplasm, actin filaments grow (dashed green arrow) and can contact the nuclear envelope. Chromosome movement is globally influenced by nuclear actin that acts in chromatin remodeler complexes and locally, at chromosome ends, by actin filaments that brush against the nuclear envelope. **(B)** Actin filaments transmit forces onto paired chromosomes during the meiotic pachytene state via LINC complexes resulting in vigorous chromosome movement. Although it was shown that nuclear actin has an influence on chromosome movement during meiosis (Lui et al., 2013), cytoskeletal actin filaments have a more drastic impact during this stage (Koszul et al., 2008).

In *S. cerevisiae*, the Rabl conformation of chromosomes is another major regulator of chromosome movement (**Figure 2**). While the centromeres are attached to the Spindle Pole Body (SPB, mammalian microtubule organizing center, spanning the yeast nuclear envelope) via nuclear microtubules, the chromosome arms reach out into the nuclear space and chromosome ends are found confined to the nuclear periphery. Furthermore, different subcompartments coexist in the nucleus that can exclude chromosomes from some of the nuclear space. For instance, the nucleolus in *S. cerevisiae* originates from rDNA on chromosome XII and is found on the opposite side of the SPB. rDNA does not seem to interact with DNA loci outside its boundaries. On the other hand, telomeres are bound to the nuclear periphery during G1 and S phase, thus, linking chromosomes to the nuclear membrane. Interestingly, telomere localization depends on its individual chromosome arm length. Telomeres on shorter chromosome arms are closer to the SPB, while telomeres on longer chromosome arms have a higher probability to be close to the nucleolus (Schober et al., 2008; Therizols et al., 2010).

Studies of subtelomeres (sequence upstream of telomeres) motion showed that the nucleolus acts as a boundary to their movement (Cabal et al., 2006; Therizols et al., 2010; Spichal et al., 2016). Furthermore, movement analyses show that the tethering of chromosomal loci to nuclear structures is mainly responsible for their different, slower, motion behavior compared to untethered loci (Hajjoul et al., 2013; Spichal et al., 2016).

A striking example is the attachment of the centromere to the SPB that confines pericentromeric chromatin movement. By detaching centromeres from the SPB using the drug nocodazole that depolymerizes microtubules, it was shown that the confinement radius of a chromosomal locus close to the centromere increases by a factor of 3 (Marshall et al., 1997) even if its speed does not change significantly (Heun et al., 2001). Centromeric DNA loci then showed a similar behavior as chromosomal loci in the middle of a chromosome arm. A detachment of the centromere from the SPB is also observed when the centromere is inactivated through transcription by the Gal1 promoter inserted close to the centromere region (Verdaasdonk et al., 2013). In this case, the same effect of increase in confinement radius of about three times has been observed. Therefore, the movement of a centromere locus depends partially on its attachment to the SPB. However, this effect decreases with increasing distance to the centromere. Recently, it was found that it is the phosphorylation of Cep3, a protein of the yeast kinetochore that is important for chromosome motion generated upon DNA damage (Strecker et al., 2016).

The speed of telomeres increases with their detachment from the nuclear membrane. This is observed in different mutants (csm4, pom152, sir4) that release telomeres from the nuclear periphery allowing telomeres to diffuse more freely as seen for other DNA loci (Hajjoul et al., 2013; Spichal et al., 2016). When telomeres are detached and statistically closer to the interior of the nucleus, their localization is still dependent on chromosome arm length. Somehow surprisingly, chromosome modeling studies could very robustly recapitulate experimental data. Centromere attachment, telomere attachment, nucleolar boundary and nuclear periphery are sufficient for telomeres and internal loci to be localized as found in experimental data (Tjong et al., 2012; Wong et al., 2012). Therefore, the environment and physical properties of chromosomes are important, if not the most important factors for their localization and hence their movement. One exception has been the analysis of telomeres in G0 state. Yeast cells will reach this state if they have to withstand periods of low nutrients. In G0, telomeres were found to come closer to the SPB while centromeres seem to be partially detached from the SPB (Laporte et al., 2013, 2016; Guidi et al., 2015; Rutledge et al., 2015). The dynamic properties of chromosomes in this particular, quiescent state, await future characterization.

## The Link Between Cytoskeleton, Nucleus, and Chromatin

First evidence for links between the cytoskeleton and chromosomes came after the observation of dramatic chromosome movements during the meiotic prophase when chromosomes start pairing. These movements are very different from chromosome movements throughout the vegetative cell cycle. They are closely linked to a complex sitting in the nuclear envelope called “LINC”, which, as its name says, connects the cytoskeleton to the nucleoskeleton. These type of complexes, conserved from yeast to men, have kept their general architecture during evolution (Rothballer and Kutay, 2013). They are composed of proteins that belong the families of SUN domain proteins (the acronym SUN is derived from Sad1p due to the conservation of the same domain in Sad1p from *S. pombe* and UNC-84 from *C. elegans* as well as KASH domain proteins (the acronym KASH is derived from the conservation of the same domain in Klarsicht from *D. melanogaster*, ANC-1 from *C. elegans*, and Syne Homology from mammals).

SUN domain proteins span the inner nuclear membrane with a transmembrane segment followed by helixes in the form of a spiral and SUN domain at the C terminus facing the nucleoplasm. KASH proteins are anchored in the outer nuclear membrane with their C-terminal part that contains the KASH domain while the N-terminal part is found in the cytoplasm (**Figure 2**). This configuration enables SUN proteins to interact with the nucleoplasm, which includes lamina in mammals, and chromatin. SUN proteins interact with KASH proteins in the intra-nuclear space of the nuclear envelope. KASH proteins are able to interact with the cytoskeleton by their cytoplasmic domain (Burke and Roux, 2009; Razafsky and Hodzic, 2009; Starr and Fridolfsson, 2010). The nuclear envelope of vertebrates contains multiple SUN and KASH proteins. Their KASH proteins Nesprine-1 and Nesprine-2 can directly interact with the actin cytoskeleton by their calponin-homology domain (CH) (Zhang et al., 2002; Padmakumar et al., 2004). Due to these protein complexes LINC complexes are also commonly called SUN-KASH nuclear-envelope bridges (Starr and Fridolfsson, 2010).

LINC proteins can influence cell shape and cell polarity by their interaction with the cytoskeleton. In metazoans, the LINC complex is also responsible for the attachment of centromeres to the nucleus, which is essential for nuclear and cellular migration (Rothballer and Kutay, 2013). The LINC complex also promote directed mobility in the particular case of damaged telomeres. Dysfunctional telomeres in MEF cells show an altered, directional and increased mobility, with an anomalous coefficient α > 1 (Lottersberger et al., 2015). Interestingly, this directed movement also depends on microtubules (disrupted in the presence of taxol) and the repair protein 53BP1. Spatial roaming by altered telomeres was shown to promote NHEJ. It was proposed that increased mobility could counteract mis-repair by disrupting aberrant connections and favoring correct ligations by NHEJ (Lottersberger et al., 2015). It will be interesting to determine if 53BP1, LINC, and microtubule connections are direct or not.

In *S. cerevisiae*, there is only one known SUN domain protein called Mps3 and there are two proteins that are potentially functional orthologues of KASH proteins, Mps2 and Csm4. It is interesting that Csm4 is a paralogue of Mps2 and that the two genes result from the same genome duplication during *S. cerevisiae* evolution. Mps3 and Mps2 are found to interact at the SPB (Jaspersen et al., 2002, 2006). This interaction is important for SPB duplication inside the membrane (Friederichs et al., 2011). An interaction of Mps3 and Csm4 has been identified, although only in meiosis. During the meiotic prophase, the observed vigorous chromosome movements are thought to be necessary for a better homology search during homolog pairing (Koszul and Kleckner, 2009). The mechanical force that drives these movements has been described to be directly generated by the actin cytoskeleton (Trelles-Sticken et al., 2000; Conrad et al., 2007, 2008; Kosaka et al., 2008; Koszul et al., 2008; Wanat et al., 2008). Mps3 binds to telomeres and the meiosis-specific protein Ndj1 via its SUN domain, while Mps3 perinuclear domain is in contact with Csm4. Csm4 also interacts with actin filaments. Actin filaments, nuclear envelope and chromosomes were followed *in vivo* thanks to fluorescent proteins that bind these structures. Nuclear protrusions of the nuclear membrane could be seen and seemed to be mediated by forces generated from actin filaments. Interestingly, chromosomes were pulled into these protrusions at the same time (Koszul et al., 2008). Evidence for chromosome-LINC-cytoskeleton connections came from the study of deletions of either Ndj1, the N-terminal domain of Mps3, Csm4 or actin filaments (by treatment with latrunculin, which inhibits actin polymerization by binding to an internal pocket of the barbed ends (Morton et al., 2000). In either of these conditions, a drastic down-regulation of chromosome movement is observed during meiosis (Trelles-Sticken et al., 2000; Conrad et al., 2007, 2008; Kosaka et al., 2008; Koszul et al., 2008; Wanat et al., 2008).

Mps3 and to a lesser extent Csm4, are also present during the vegetative cell cycle. In this cell cycle phase, Mps3 was shown to localize telomeres to the nuclear envelope during S-phase (Schober et al., 2009). The absence of Csm4 also provokes subtelomere delocalization away from the nuclear envelope (Spichal et al., 2016). This is rather unexpected since a direct interaction of Csm4 with subtelomeres was not anticipated, however, a general change in nuclear envelope structure could account for this result. Furthermore, it was shown that actin filaments inhibition by the drug Latrunculin could further influence chromosome movement and localization even in the absence of Csm4. This suggested that the LINC complex acts differently during the vegetative cell cycle than during meiosis (Spichal et al., 2016). Besides, it was found that, if fusing actin to a nuclear pore protein through Actin binding domain of LifeAct, the interactions of actin and the nuclear envelope increased and the nuclear envelope showed deformations. This artificial actin filament binding to the nuclear envelope increased both nuclear envelope diffusion dynamics and telomere dynamics at the nuclear envelope but not of chromosome loci in the middle of the chromosome arms (Spichal et al., 2016). This suggests that the increase in dynamics observed does not result from movements of the nucleus, but that actin filaments also have the capacity to influence chromatin that is close to the nuclear periphery during the vegetative cell cycle.

While the nuclear envelope seems to have an influence on the mobility of chromatin tethered to nuclear periphery, the reverse—chromatin can influence nuclear envelope mobility—also seems to be true. Schreiner et al. (2015) report that nuclear envelope rigidity is compromised in fission yeast *Saccharomyces pombe* cells lacking lamins and chromatin attachment. In *S. pombe*, nucleo-cytoskeleton interactions are mediated by microtubules. The authors show that cytoplasmic microtubules can deform the nuclear membrane, while actin filaments only cause minor membrane deformations. This nuclear envelope deformability is increased if proteins responsible for the attachment of chromatin to the inner nuclear membrane are mutated emphasizing the dependence of the nuclear envelope stiffening to its links with chromatin.

## Proteins from Cytoskeleton in the Nucleus: What Role in Chromosome Motion?

By definition the cytoskeleton exists in the cytoplasm. Yet, various components and proteins of the cytoskeleton have been found in the nucleus. In there, they play different roles as compared to their cytoplasmic function. Cytoskeletal proteins discovered in the nucleus are involved in chromatin remodeling, transcription and nuclear transport. Several of these proteins have been implicated in chromosome movement. The different types of proteins include myosins (proteins that ‘walk’ on actin filaments in the cytoplasm), actin related proteins (ARPs), actin itself, lamin and tubulin.

Actin and proteins similar to actin in sequence, called ARPs (Actin Related Proteins) are found in large chromatin remodeler complexes. ARPs, conserved from yeast to humans, do not have an ATPase activity. *S. cerevisiae* has 10 ARPs that are named according to their level of sequence similarity with actin (Poch and Winsor, 1997). Arp1-Arp3 and Arp10 are mainly found in the cytoplasm, while Arp4-Arp9 are nuclear. *S. cerevisiae* possesses 5 chromatin remodelers that contain ARPs and/or actin; INO80, SWR1, NuA4, SWI/SNF and RSC (Dion et al., 2010). It was observed that a mutation in the subdomain 2 of actin (act1-2) decreases the capacity of INO80 to bind to nucleosomes and to mobilize them, while a different mutation (act1-1) that affects the polymerization of actin does not have an effect. Thus, in yeast, when actin interacts with DNA, it is by its pointed end and actin likely does not polymerize (Kapoor et al., 2013). Even though the affinity of actin for DNA is higher than that of its subunit Ino80, Arp4 and Arp8, and other proteins might also contribute to this interaction. The principal role of actin in this complex might be limited to its ATPase activity that induces a conformational change in the complex INO80 during its interaction with chromatin (Kapoor and Shen, 2014).

ARP mutants as well as mutants of other subunits of chromatin remodelers have different phenotypes that include transcriptional deregulation, errors in DNA replication and repair (Papamichos-Chronakis and Peterson, 2008; Shimada et al., 2008; Vincent et al., 2008). However, whether these defects are related to defective chromatin dynamics remains to be determined. Besides ARP proteins, nuclear actin itself has been directly implicated in gene expression regulation. Sharili et al. (2016) report that NLS-actin expressed in human keratinocytes could target multiple cytoskeletal genes that were down-regulated under high levels of nuclear actin. Actin that accumulated in the nucleus changed adhesive and focal cohesion organization and stopped cell motility. Furthermore, when actin was inhibited from entering the nucleus, by knocking down Importin 9, cell migration was enhanced. Hence, the actin cytoskeleton and nuclear actin seem to be in an equilibrium, which connects gene expression to cell mobility. It is an interesting idea that a rapid decomposition of the actin cytoskeleton could directly influence gene expression by a sudden influx of actin monomers into the nucleus. Recently, the importance of nuclear actin has become clearer as actin is required for chromosome movement. It was also shown that actin is required for DNA repair, although it could also be part of the repair machinery (Spichal et al., 2016). Likewise, nuclear actin is equally thought to contribute to chromosome dynamics during the meiotic prophase, even though actin cytoskeleton-driven movement seems to be predominant (Lui et al., 2013). Filamentous nuclear actin was also observed in many cells of multicellular organism (Hendzel, 2014). However, no evidence for filamentous actin has been found in budding yeast so far.

Nuclear actin and myosin I were both shown to be necessary for the functional organization of the nucleus in mammalian cells. In the cytoplasm, myosins are motor proteins that associate with actin and use ATP hydrolysis to drive muscle contraction, cell motility and organelle movement. In order to do so, myosin requires filamentous actin (Sellers, 2004). On the other hand, early replicating chromosomes are in the nuclear center while late-replicating heterochromatic regions are found at the nuclear periphery (Spector, 2003). It is striking that chromosomal locus repositioning is an active process that requires nuclear actin and nuclear myosin 1 (NM1), pointing to the nuclear role of these cytoskeletal proteins in chromosome motion (Chuang et al., 2006; Dundr et al., 2007). Likewise, nuclear motor proteins like actin and myosin have been shown to move whole chromosome territories in response to serum starvation in primary human fibroblasts (Mehta et al., 2010). It was found that the motor function of NM1 is necessary for its chromatin recruitment and also to relocalize chromosome territories after DNA damage in human fibroblasts. Hence, NM1 was proposed to guide DNA damage induced chromosome territory relocation (Kulashreshtha et al., 2016). Moreover, NM1 has been associated with various functions in chromatin remodeling and transcription, similar to nuclear actin. In particular, NM1 was shown to interact with RNA polymerase I and ribonucleoproteins in mammals and to be required for elongation and ribosomal RNA maturation, a process that might require chromosome motion to be efficient (Fomproix and Percipalle, 2004; Philimonenko et al., 2004; Obrdlik et al., 2010).

In multicellular organisms, lamina might also play an important role in chromosome motion. Lamins are part of a nuclear matrix, the nucleoskeleton, absent from yeasts. The nucleoskeleton is a meshwork of intermediate filaments on the nuclear side of the nuclear periphery that helps to keep the nuclear architecture in place. It is interesting to note that, while telomere or centromere diffusion was shown to be anomalous (α ranging from 0,4 to 0,7), diffusion of telomeres in MEF cells depleted for Lamin A, became normal with α = 1 (Bronstein et al., 2009). This result is surprising since telomeres have not been found to be attached to the nuclear periphery. Thus, absence of Lamin A could alter genome dynamics from slow anomalous diffusion to fast and normal diffusion, through interaction taking place not only at the nuclear periphery but also in the nuclear interior (Bronshtein et al., 2015). It is possible that these additional restraints are necessary to keep especially large genomes in place. Future studies will establish whether Lamin A affects chromatin motion directly or whether it acts through other structural proteins. Furthermore, it is noteworthy that anomalous chromosome dynamics are seldom directly associated with human disease. Harmful chromosomal rearrangements have often been observed in cancer as consequences of potentially aberrant dynamics and localization (Hasty and Montagna, 2014). Furthermore, multiple mutants of the LINC complex have been associated with neurological disorders in mice, that has been related to deafness (Starr and Fridolfsson, 2010). It is likely that mutations of the LINC complex have influences on chromosome dynamics in the interior of the nucleus. Mehta et al. (2011) describe that a treatment of proliferating fibroblasts derived from Hutchinson–Gilford progeria syndrome (HGPS) patients could restore normal chromosome territory localization and dynamics. HGPS leads to premature aging in children caused by the mutation in the A-type lamin gene LMNA. This leads to the expression of a truncated form of lamin A, progerin, that remains farsenylated. The authors show that a treatment with a farnsyltransferase inhibitor leads to a less toxic protein and reestablishes chromosome territory position and dynamics observed in wt cells (Mehta et al., 2011). Further future studies might use chromosome dynamics as a tool to identify early misregulations and treatment of this could lead to an early prevention of the associated diseases.

## Conclusion

Chromosome movement is a complex process that is regulated by different mechanisms. Chromosomes do not only move in cell division but chromosome movement is required for all sorts of different regulations and cellular mechanisms in all cell cycle stages.

Chromosome dynamics are characterized by the nature of the DNA fiber and the chromatin structure. The physical properties of the chromosome as well as its immediate environment inside the nucleus determine the diffusion behavior of chromosomal loci. The nature of diffusion of different chromosomal loci has been shown to be confined and/or subdiffusive or anomalous. The disagreement between the different studies results from different methods and conditions used. Overall it is interesting to note that the type of diffusion helps to understand how chromosome diffusion is driven and how chromatin properties are challenged.

Chromosome movement also seems to be tightly linked to the cytoskeleton and individual proteins thereof found inside the nucleus. The relationship between chromosome movement and the cytoskeleton has been best characterized for the actin cytoskeleton. Actin filaments interact with the nuclear envelope to mediate interactions with chromatin. The direct interaction of the cytoskeleton with chromatin via the nuclear envelope can influence chromosome dynamics most prominently during meiosis via LINC complexes, but interactions have also been found during the vegetative cell cycle. As it was shown that a rapid increase in nuclear actin, for example through actin filament depolymerization, can influence gene expression in mammalian cells (Dopie et al., 2012; Rajakylä and Vartiainen, 2014), it is likely that there exists an equilibrium between actin cytoskeleton and nuclear actin that acts on the tightly linked processes of chromosome dynamics and gene expression.

The universal characteristic of skeleton forming proteins to equally act on DNA metabolism seems to be remarkable, however, the exact mechanisms that regulate the signaling and equilibrium between the cytoplasmic and nuclear pools still need exciting and challenging research to be determined.

## Author Contributions

MS and EF contributed substantially to the conception and design of the work; and drafted the work; and approved the version to be published; and agreed to be accountable for all aspects of the work in ensuring that questions related to the accuracy or integrity of any part of the work are appropriately investigated and resolved.

## Conflict of Interest Statement

The authors declare that the research was conducted in the absence of any commercial or financial relationships that could be construed as a potential conflict of interest. The reviewer KB declared a shared affiliation, though no other collaboration, with one of the authors MS to the handling Editor, who ensured that the process nevertheless met the standards of a fair and objective review.
